# Src Tyrosine Kinase Inhibitory and Antioxidant Activity of Black Chokeberry and Bilberry Fruit Extracts Rich in Chlorogenic Acid

**DOI:** 10.3390/ijms242115512

**Published:** 2023-10-24

**Authors:** Sanda Vladimir-Knežević, Maja Bival Štefan, Biljana Blažeković, Dubravko Jelić, Tea Petković, Marta Mandić, Ekaterina Šprajc, Sandy Lovković

**Affiliations:** 1 Department of Pharmacognosy, Faculty of Pharmacy and Biochemistry, University of Zagreb, 10000 Zagreb, Croatia; svladimir@pharma.hr (S.V.-K.); bblazekovic@pharma.hr (B.B.); tpetkovic@pharma.hr (T.P.); 2 Selvita Ltd., 10000 Zagreb, Croatia; dubravko.jelic@gmail.com; 3 Faculty of Pharmacy, University of Mostar, 88000 Mostar, Bosnia and Herzegovina; marta.mandic@farf.sum.ba; 4 Jamnica Plus Ltd., 10000 Zagreb, Croatia

**Keywords:** *Aronia melanocarpa*, *Vaccinium myrtillus*, black chokeberry, bilberry, chlorogenic acid, Src tyrosine kinase inhibitory activity, antioxidant activity

## Abstract

Edible berries such as the fruits of black chokeberry (*Aronia melanocarpa* (Michx.) Elliott) and bilberry (*Vaccinium myrtillus* L.) are considered to be rich in phenolic compounds, which are nowadays attracting great interest due to their promising health benefits. The main objective of our study was to investigate, for the first time, their inhibitory properties on Src tyrosine kinase activity, as this enzyme plays an important role in multiple cellular processes and is activated in both cancer and inflammatory cells. In hydroethanolic fruit extracts, 5.0–5.9% of total polyphenols were determined spectrophotometrically, including high amounts of hydroxycinnamic acid derivatives. HPLC analysis revealed that the black chokeberry and bilberry extracts contained 2.05 mg/g and 2.54 mg/g of chlorogenic acid, respectively. Using a time-resolved fluorescence resonance energy transfer (TR-FRET) assay, the extracts studied were found to have comparable inhibitory effects on Src tyrosine kinase, with IC_50_ values of 366 µg/mL and 369 µg/mL, respectively. The results also indicated that chlorogenic acid contributes significantly to the observed effect. In addition, both fruit extracts exhibited antioxidant activity by scavenging DPPH and NO radicals with SC_50_ values of 153–352 µg/mL. Our study suggested that black chokeberry and bilberry fruits may be beneficial in cancer and other inflammation-related diseases.

## 1. Introduction

Berry fruits, their processed foods, and dietary supplements have become popular as a result of their promising health benefits. In addition to valuable nutrients, dark-colored berries are rich in bioactive compounds, of which the structurally diverse phenolic compounds are the most abundant. Numerous studies have shown that polyphenol-rich berries possess a broad spectrum of biological and pharmacological activities that have a positive effect on aging and age-related chronic diseases [1,2]. 

Black chokeberry (*Aronia melanocarpa* (Michx.) Elliott) of the family Rosaceae is a perennial shrub native to eastern North America. It is now widely cultivated in many European countries, but its fruit is rarely eaten fresh and unprocessed because of its tart and sour-bitter taste. So, the fruits are often used to make various food products including syrups, juices, jellies, alcoholic and energy drinks, teas, and dietary supplements. Native Americans used to treat colds with the fruit of black chokeberry [3]. Today, the fruit and flowers are known as traditional remedies for flu and to strengthen the immune system [4]. Black chokeberry is also used for hypertension and atherosclerosis [5]. It has one of the most polyphenol-rich berry fruits, with a high content of proanthocyanidins, anthocyanins, and phenolic acids, while flavonoids form the smallest class of phenolic compounds [3,4]. It is estimated that fresh fruits contain up to 3 g of polyphenols per 100 g [6]. High polyphenol content has been associated with a variety of health-promoting effects, including anti-inflammatory, antioxidant, immunomodulatory, anticarcinogenic, antimicrobial, antidiabetic, cardioprotective, liver-protective, and neuroprotective effects [7,8,9,10,11,12,13].

Bilberry (*Vaccinium myrtillus* L.) is a wild-growing shrub that belongs to the Erica-ceae family. It is native to Eurasia and, unlike black chokeberry, is not cultivated. The pleasant, sweet, and slightly astringent taste of the fruit makes it very popular as a fresh fruit and processed food or as a dietary supplement. Based on long-standing traditional use, the dried ripe fruit of bilberry (Myrtilli fructus siccus) is considered a medicinal product for the treatment of mild diarrhea and inflammation of the oral mucosa, while the fresh ripe fruit (Myrtilli fructus recens) is recommended for the treatment of capillary fragility and symptoms of venous insufficiency [14,15]. Bilberry fruit is a rich source of biologically active compounds, especially phenolic compounds such as anthocyanins, hydroxycinnamic acid derivatives, flavonoids, and proanthocyanidins [16,17,18]. They exhibit anti-inflammatory activity, which could translate into potential preventive and therapeutic effects in metabolic syndrome, cancer, diabetes, and cardiovascular, ophthalmic, and neurological diseases [19,20].

Both black chokeberry and bilberry fruits contain high levels of hydroxycinnamic acids, of which chlorogenic acid is the most abundant. This is an ester of caffeic acid and quinic acid, that is widely distributed in plants. We consume the majority of chlorogenic acid from coffee, but it is also found in various herbs, fruits, and vegetables that we have in our diet [21,22]. Previous studies have shown its multiple biological and pharmacological effects, including antioxidant, anti-inflammatory, anticancer, antihyperlipidemic, antidiabetic, antihypertensive, and antineurodegenerative effects [23]. To exert their functions, chlorogenic acid and other phenolic compounds target various molecular mechanisms and signaling pathways. If we consider ROS-induced oxidative damage and various pathologies in different organ systems, it is clear that oxidative stress leads to the development of various disorders and aging processes [24]. By interacting with nucleic acids (DNA damage and strand breaks), lipids (lipid peroxidation), and proteins, especially cysteine residues, ROS can cause cell toxicity. ROS also has effects on several signaling pathways, such as modulation of NF-κB/Nrf2 activation, which plays an important role in regulating cellular responses to oxidative stress and inflammation [25]. Numerous studies have demonstrated that bioactive phenolic compounds in berries may act as ROS radical scavengers by donating electrons to free radicals, thus protecting DNA, proteins, and lipids from oxidative damage. Some systematic reviews and meta-analyses of clinical studies have described the health benefits of certain berry species and phenolic compounds on cardiovascular diseases, metabolic disorders, and cancer. However, future research is needed to clarify the mechanisms of action of the bioactive berry constituents and their health benefits [26]. 

Most signal transduction pathways in humans are regulated by protein kinases through the phosphorylation of their protein substrates. Protein phosphorylation is an important cellular regulatory mechanism as many enzymes and receptors are activated/deactivated by it [27,28]. The Src family of non-receptor tyrosine kinases in humans comprises eleven members, of which Src, Yes, and Fyn are present in almost all cells, whereas the other members are restricted to specific tissues and organs [29]. Src tyrosine kinases phosphorylate tyrosine residues on target proteins and are thus involved in the regulation of cellular signaling pathways that control numerous processes important for the maintenance of cellular homeostasis and survival [30]. To activate a tyrosine kinase receptor, key amino acids must be in the correct positions to facilitate phosphate group transfer, and the peptide-substrate binding site must be accessible [31]. Src tyrosine kinases play an important role in carcinogenesis, as their excessive activation and expression lead to cell proliferation disorders associated with tumor invasion, metastasis, and angiogenesis [30,32]. Recent scientific evidence also points to the role of the Src kinase family in the development of inflammation-related diseases, as they also control the transmission of signals associated with the inflammatory response [33,34,35].

Because of the integral role of protein kinases in the regulation of intracellular homeostasis and the transduction of extracellular signals, dysregulation of their activity is directly related to numerous progressive diseases. Therefore, small molecules that inhibit protein kinases are now among the most-studied drug classes for the treatment of various diseases such as cancer and inflammatory and autoimmune diseases [36,37]. Given their enormous therapeutic potential and drug-like properties, kinase inhibitors are a rapidly growing and important category of target therapeutics. Most protein kinase inhibitors already approved or about to be approved for clinical use, as well as most kinase inhibitors currently in clinical trials, target tyrosine kinases. Among these kinase inhibitors, those derived from various plant sources are also described in the literature. They belong to different classes of compounds, including polyphenols [38]. 

With this background, we aimed to evaluate the antioxidant properties of black chokeberry and bilberry fruit extracts and, for the first time, to investigate their ability to inhibit Src tyrosine kinase activity. Since chlorogenic acid is widely distributed in plants and is one of the most important phenolic acids in the human diet, it is intensively studied for its health-promoting activities [23]. However, the role of chlorogenic acid as an inhibitor of Src kinases or as a scavenger of NO has not yet been investigated. Therefore, our further objective was to evaluate the role of chlorogenic acid, one of the most abundant phenolic constituents of black chokeberry and bilberry fruits, in the biological effects of interest. 

## 2. Results and Discussion

### 2.1. Content of Polyphenols in Black Chokeberry and Bilberry Fruit Extracts

The contents of total polyphenols, and the individual proportions of the various polyphenol groups in the hydroethanolic fruit extracts, were determined spectrophotometrically. Table 1 shows that both the black chokeberry and bilberry extracts were rich in phenolic constituents (5.90% and 4.96%, respectively). The most abundant compounds in both extracts studied were phenolic acids. The black chokeberry extract contained a high 4.09% of hydroxycinnamic acids, while the percentage in the blueberry extract was 1.51%. Anthocyanins were also present in considerable amounts in the fruits of chokeberry (0.41%) and blueberry (1.06%), while the percentage of flavonoids was much lower (0.17% and 0.12%, respectively). 

Our study confirms previous findings on the high content of polyphenols in the fruit, juice, and extracts of black chokeberry [5,39,40]. A review of literature data shows that phenolic compounds in black chokeberry have been extensively studied. Among anthocyanins, cyanidin glycosides were identified as particularly representative, with cyanidin-3-galactoside predominating. Fruits were found to contain large amounts of procyanidins composed of flavan-3-ol monomers, mainly epicatechin. The glycosides of quercetin were identified to be the most abundant flavonoids. In addition, previous studies have shown that the fruit of black chokeberry is characterized by a high content of phenolic acids, mainly chlorogenic acid and neochlorogenic acid [22,41,42]. 

The results of spectrophotometric determination of total polyphenols and individual polyphenol groups in the bilberry extract are in agreement with previous studies on bilberry fruit [43,44,45]. The bilberry extract had a slightly lower total polyphenol content than black chokeberry. However, the bilberry-fruit extract contained about 2.5 times more anthocyanins but less phenolic acids. Literature studies have shown that chlorogenic acid and glycosides of cyanidin, delphinidin, and quercetin are the predominant single phenolic constituents of bilberry fruit [45,46].

### 2.2. Content of Phenolic Acids and Flavonoids in Black Chokeberry and Bilberry Fruit Extracts

**Figure 1:**
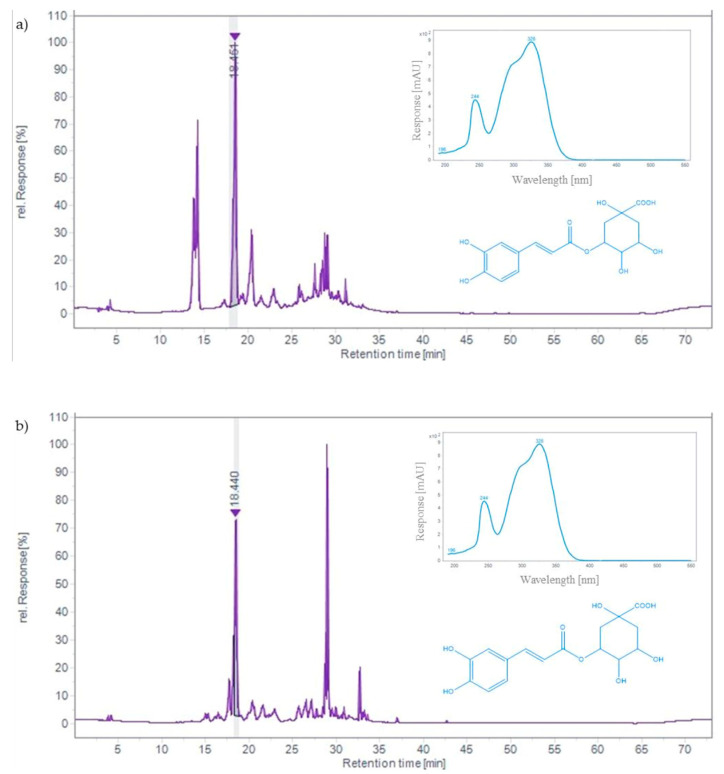
HPLC chromatograms of hydroethanolic extracts of black chokeberry fruits (**a**) and bilberry fruits (**b**) with UV-spectrum of chlorogenic acid recorded at 320 nm.

TLC analysis revealed that chlorogenic acid was the main phenolic compound in both the chokeberry and bilberry extracts. In addition to chlorogenic acid, black chokeberry also contained a significant amount of neochlorogenic acid, which was not the case for the bilberry. As can be seen in the HPLC chromatograms shown in Figure 1, the chlorogenic acid peak in both samples stands out, with a retention time of 18.44 min. The extract from black chokeberry fruit contained 2.05 mg/g of chlorogenic acid, while 2.54 mg/g of chlorogenic acid was determined in bilberry-fruit extract. Several previous studies have also demonstrated high levels of chlorogenic acid in black chokeberry fruit. For example, about 1 mg/g of chlorogenic acid was determined in a German sample [47]. In fresh fruits from Russia and Belarus, it was identified as the main hydroxycinnamic acid in different varieties (0.2–0.8 mg/g), accounting for more than 50% of the total content [41]. Research on samples from Poland showed that, as the fruit ripens, the proportion of chlorogenic acid decreases in favor of anthocyanins [22]. In light of the results obtained for bilberry, similar levels of chlorogenic acid were found in the extracts of bilberry fruit from Romania and Montenegro [17,45]. Our results confirmed that the hydroethanolic extracts of black chokeberry and bilberry are rich natural sources of chlorogenic acid, which is considered an essential component of their health benefits, including antioxidant, anti-inflammatory, anticarcinogenic, antidiabetic, and cardioprotective properties [22]. 

In addition to chlorogenic acid, neochlorogenic acid was also detected in black chokeberry fruit, while bilberry fruit did not contain it. This finding is reflected in the HPLC chromatograms (Figure 1), where the peak of neochlorogenic acid (Rt = 14.12) is seen only in the extract of black chokeberry. Our results showed that the extract contained a high amount of neochlorogenic acid (2.07 mg/g), which was comparable to chlorogenic acid (Table 2).

Previous studies also support the presence of neochlorogenic acid in black chokeberry fruit. Kaloudi et al. [48] detected neochlorogenic acid and chlorogenic acid in fresh fruits from Greece in a ratio of 1:1.8. Neochlorogenic acid was also determined in the fresh fruits of five chokeberry cultivars from Belarus, Russia, and Denmark. Its content varied between 0.15 and 0.46 mg/g, being 1.3–1.8 times lower than that of chlorogenic acid [41]. Quercetin glycosides were found to be the major flavonoid compounds in the fruits of black chokeberry and bilberry. The results summarized in Table 2 show that bilberry contains the highest amount of isoquercitrin (1.36 mg/g). This 3-*O*-glucoside of quercetin was also present in the extract of black chokeberry but in a lower amount (0.67 mg/g), along with a significant amount of 3-*O*-galactoside (0.50 mg/g) and 3-*O*-rutinoside (0.34 mg/g). Our results are consistent with previous reports of the highest content of quercetin compounds among flavonoids in the fruits of black chokeberry [49] and blueberry [49,50].

### 2.3. Src Tyrosine Kinase Inhibitory Activity of Black Chokeberry and Bilberry Fruit Extracts

Src tyrosine kinase (Src) is the first proto-oncogene tyrosine kinase ever described. Both expression and activation of Src are enhanced in various human cancers and correlate with malignancy progression and the development of metastasis and invasion. The role of Src in oncogenesis has led to the discovery of other members of the Src protein kinase family and the search for cancer therapies. Because of increasing evidence of its crucial role in tumor progression, Src has emerged as a promising target for anticancer therapy [51,52]. Src is primarily involved in numerous signaling pathways that regulate and maintain cell metabolism, cell contact, and cell migration. It also controls signal transduction associated with inflammatory responses [53]. A previous study reported that Src triggers macrophage-mediated inflammatory responses. Various inflammatory diseases, such as rheumatoid arthritis, atherosclerosis, cancer, obesity, and diabetes, are closely related to macrophage activation [54]. In recent years, numerous natural products have been described as starting points for new chemical compounds that act as protein kinase inhibitors and are potential drug candidates [55]. 

**Figure 2:**
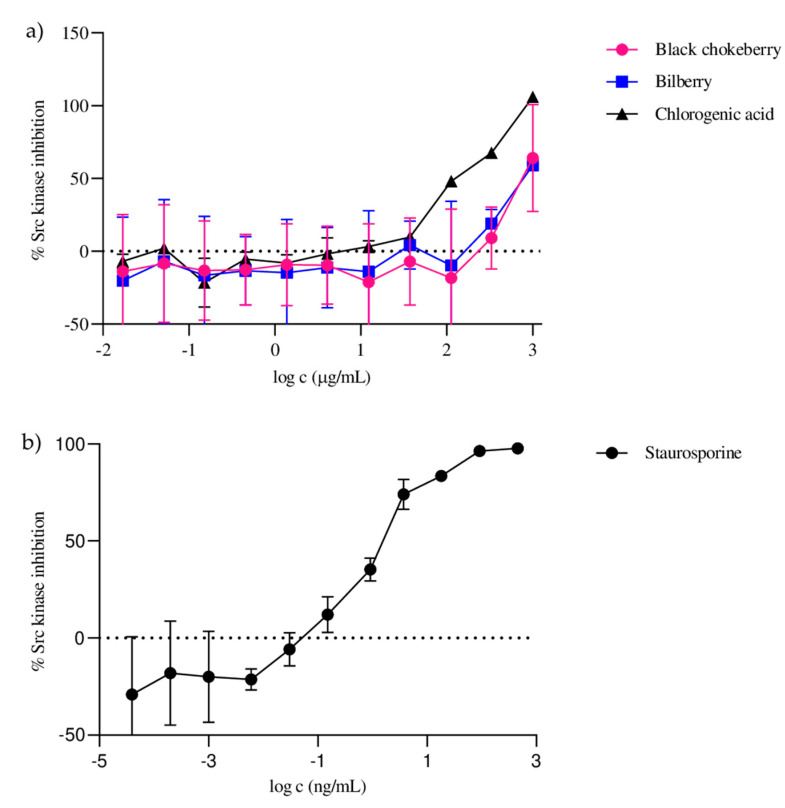
Dose–response curves of Src kinase inhibition for black chokeberry fruits, bilberry fruits, chlorogenic acid (**a**) and staurosporine (**b**).

The present study evaluated black chokeberry and bilberry fruit hydroethanolic extracts for their inhibitory effect on Src activity using a time-resolved fluorescence resonance energy transfer assay (TR-FRET), with staurosporine serving as a positive control (IC_50_ = 0.70 ± 0.05 ng/mL). The studied extracts inhibited Src activity in a dose-dependent manner (Figure 2) and showed a comparatively significant inhibitory effect, with IC_50_ values of 366 ± 17.02 µg/mL and 369 ± 21.64 µg/mL, respectively. Inhibition of Src activity by chlorogenic acid, one of the most abundant constituents of the extracts studied, was also tested. The results showed that chlorogenic acid significantly contributed to the inhibition of Src activity, with an IC_50_ value of 122 ± 9.13 µg/mL, which was a three-times-stronger activity than that of the extracts studied. The primary responsibility of chlorogenic acid for the inhibitory effects of the extracts is also reflected in the result that black chokeberry and bilberry extracts with approximately equal amounts of chlorogenic acid showed very similar inhibitory effects on Src activity.

These are the first data on the inhibitory effects of chokeberry and bilberry on Src activity, helping to clarify the mechanisms of action by which they may exert anticancer and anti-inflammatory effects. Previously, black chokeberry extracts and their isolated bioactive components have shown growth-inhibitory effects on various human cancer cells, including HT-29 and Caco-2 colon cancer, MCF-7 and MDA-MB-231 breast cancer, A549 and H1299 non-small-cell lung cancer, and BGC-803 gastric cancer [9,56]. They have been found to inhibit the cell proliferation of cancer cells and arrest the cell cycle through various mechanisms of action, such as reducing the expression of cyclin A and B genes [57], upregulating the tumor suppressor carcinoembryonic antigen-related cell adhesion molecule 1 [58], inducing c-Myc protein degradation [59], decreasing the stability of β-catenin, and thus inhibiting the expression of related proteins in the Wnt/β-catenin signaling pathway [60]. The anticancer activity of bilberry has also been the subject of previous studies demonstrating its antiproliferative and proapoptotic properties [61]. Bilberry-fruit extract and its polyphenol fraction have shown an anticancer effect on hormone-dependent (LNCaP) and hormone-independent (PC3 and DU-145) prostate cancer cell lines [62], and HSC-3 oral carcinoma cells [63]. The growth and invasive potential of human H1229 non-small-cell lung cancer cells were suppressed by bilberry anthocyanins [64], which were found to induce redox-sensitive caspase 3-related apoptosis in B-cell chronic lymphocytic leukemia through dysregulation of the Bcl-2/Bad pathway [65]. Bilberry was also able to inhibit proliferation and induce apoptosis in MCF7 breast cancer cells via a mechanism that has no effect on microtubules or mitosis at the lowest effective concentrations, while microtubule organization was affected by the higher concentrations [66]. Studies on colorectal cancer indicated that bilberry inhibited intestinal tumor formation and cancer cell growth, which was confirmed by an uncontrolled pilot human study [67].

Many studies have shown a close relationship between cancer and chronic inflammation [68]. Src is overactive in both cancer cells and immune cells that infiltrate tumors. It is also involved in cytokine-mediated communication between cancer and inflammatory cells [69]. Therefore, inhibition of Src activity is certainly an important mechanism to prevent chronic inflammation and thus the occurrence of cancer and other diseases associated with inflammation. Accordingly, our results demonstrated the inhibitory potential of black chokeberry and bilberry. In terms of their anti-inflammatory properties, previous studies have shown that black chokeberry inhibits the release of inflammatory markers such as tumor necrosis factor-α (TNF-α), interleukin (IL)-1β, and IL-8, as well as the activation of nuclear factor-kappa B (NF-κB) [6,70]. Bilberry has also been found to exert anti-inflammatory effects via multiple mechanisms of action, including reducing the expression of TNF-α, IL-6, and IL-1β, inducing nitric oxide synthases and cyclooxygenases, and altering the NF-κB and Janus kinase signal transducer and activator of transcription signaling pathways [71]. 

Our findings extend the knowledge of the potent biological activity of chlorogenic acid and are consistent with previous results indicating its great potential in the prevention and treatment of various types of cancer and inflammatory diseases. For example, a recent review indicated that chlorogenic acid has excellent protective effects against various liver diseases associated with different signaling pathways, including AMP-activated protein kinase (AMPK) and extracellular signal-regulated kinases 1 and 2 (ERK1/2) [72]. Zhou et al. [73] demonstrated that chlorogenic acid has an anti-glioma effect on U373 cells by downregulating the SRC/MAPKs signaling pathway. Chlorogenic acid was also found to inhibit Bcr-Abl tyrosine kinase and trigger p38 mitogen-activated protein kinase-dependent apoptosis in chronic myelogenous leukemia cells [74]. An isomer of chlorogenic acid, 3-*O*-caffeoylquinic acid has been shown to regulate lipopolysaccharide-induced TNF-α production in microglia, deactivate c-Src and abrogate c-Src activation during proinflammatory microglia stimulation, which prevents ROS generation in these cells [75]. In addition to in vitro evaluation and animal models, chlorogenic acid has also been tested in a clinical setting. A phase I trial of chlorogenic acid injection demonstrated clinical benefits for patients with high-grade glioma, who had relapsed after previous standard therapies, and a favorable safety profile up to a dose of 5.5 mg/kg [76]. 

### 2.4. Antioxidant Activity of Black Chokeberry and Bilberry Fruit Extracts 

The antioxidant activity of the fruit extracts at concentrations of 6.25–800 µg/mL was evaluated in comparison with chlorogenic acid using two different assays. The DPPH assay is commonly used to evaluate the ability of natural products to scavenge free radicals. Unlike the DPPH radical, a stable synthetic free radical, NO is an important signaling molecule involved in the regulation of various physiological processes such as neurotransmission, smooth muscle relaxation, blood pressure regulation, vasodilation, defensive mechanisms, cell function, and control of inflammatory and immunological responses. However, when NO is formed in excessive amounts, it can react with superoxide anions to form peroxynitrite anion, a very damaging species for proteins, lipids, and DNA [77,78] Therefore, the ability of plant extracts and their pure constituents to directly scavenge NO may be useful in preventing pathological conditions associated with oxidative stress. Both studied fruit extracts showed a concentration-dependent ability to scavenge DPPH and NO radicals (Table 3 and Table 4). In both cases, black chokeberry was a more effective radical scavenger than bilberry, whose percent inhibition values were consistently lower. At the lowest concentrations tested, chlorogenic acid had an effect similar to that of the extracts at concentrations of 50–100 µg/mL. The ability of chlorogenic acid to inhibit 90% of DPPH radicals was already achieved at a concentration of 100 µg/mL, but at the highest concentration tested, there was no significant difference compared to black chokeberry, while the effect was slightly higher compared to bilberry. 

Black chokeberry also lagged behind chlorogenic acid when tested for its ability to scavenge NO radicals at lower concentrations. However, at the highest concentration tested, black chokeberry showed better efficacy. It scavenged more than 90% of the NO radicals generated, while chlorogenic acid scavenged less than 80% of the radicals. The bilberry extract at the highest concentration tested achieved an effect of slightly more than 60%, again indicating a weaker antioxidant effect compared to the black chokeberry extract. 

The DPPH and NO radical scavenging activities of the tested extracts and chlorogenic acid were evaluated using SC_50_ values, which represent the concentrations of the tested sample required to scavenge the original radical concentration by 50%. The results are presented in Table 5. The lowest SC_50_ value of 18.39 µg/mL and 20.41 µg/mL obtained for chlorogenic acid showed the highest ability to scavenge DPPH and NO radicals, respectively. Black chokeberry extract has stronger antioxidant properties than bilberry extract, as the SC_50_ values are twice as low. Regardless of the fact that two completely different radicals are used, it is interesting to note that chlorogenic acid has the same antioxidant activity, as evidenced by very similar SC_50_ values. Moreover, the same is true for the fruit extracts tested. 

The presented results showing the strong antioxidant activity of the hydroethanolic extracts of the two tested fruits are in agreement with the data in the literature [42,61]. Compared to other small dark fruits, black chokeberry is generally considered to have a stronger radical-scavenging activity due to its higher content of phenolic compounds [3]. The antioxidant activity of black chokeberry and bilberry has been confirmed in various tests such as the DPPH radical-scavenging assay [3,42,61], which is the most commonly used. Data on their ability to scavenge NO radicals, however, are scarce. Denev et al. [47] demonstrated the antioxidant activity of anthocyanin-rich extracts of bilberry and black chokeberry via the electrochemical measurement of NO radicals. Kolarov et al. [79] showed that bilberry fruit extract has a high ability to scavenge NO radicals, which correlates with the content of anthocyanins and flavan-3-ols, but they did not investigate phenolic acids and their influence on the antioxidant activity of bilberry fruits. Bilberry was also found to suppress nitric oxide (NO) and pro-inflammatory cytokine formation in LPS-induced RAW 264.7 cells and to exhibit antioxidant and anti-inflammatory properties in vitro [80]. Accordingly, our study confirmed the high capability of the tested fruit extracts to scavenge free radicals and thus prevent oxidative stress, which contributes significantly to the etiology of various chronic diseases. 

## 3. Materials and Methods

### 3.1. Chemicals 

Adenosine 5′-triphosphate disodium salt dehydrate (ATP), anhydrous sodium sulfate, aluminium chloride, 2-aminoethyl diphenylborinate, acetonitrile (HPLC grade), chlorogenic acid (95%), 2,2-diphenyl-1-picrylhydrazyl (DPPH), dimethyl sulfoxide (DMSO), ethyl acetate, ethylenediaminetetraacetic acid (EDTA), ethylene glycol tetraacetic acid (EGTA), gallic acid (95%), 4-(2-hydroxyethyl)-1-piperazineethanesulfonic acid (HEPES), hyperoside, isoquercitrin, magnesium chloride (MgCl_2_), neochlorogenic acid, phosphate buffer saline (PBS), polyethylene glycol 4000 (PEG 4000), rutin, sulphanilamide, staurosporine and Tween 20 were purchased from Sigma-Aldrich (St. Louis, MO, USA). Src (08-173) was obtained from Carna Bioscience (Natic, MA, USA). LANCE Ultra ULight-TK peptide (TRF0127-M), LANCE detection buffer 10× (CR97-100), LANCE europium-labeled anti-phosphotyrosine (PT66) antibody were purchased from Perkin Elmer (Waltham, MA, USA). Dithiothreitol (DTT) was purchased from Bio-Rad (Hercules, CA, USA). N-(1-naphthyl) ethylenediamine dihydrochloride (NED), sodium molybdate and sodium nitrite were provided by Fluka (Buchs, Switzerland). Acetic, formic, hydrochloric and phosphoric acids were obtained from Kemika (Zagreb, Croatia). Aceton, ethanol, methanol and sodium carbonate decahydrate were purchased from Gram-Mol (Zagreb, Croatia). Folin-Ciocalteau’s phenol reagent, and sodium nitroprusside were purchased from Merck (Darmstadt, Germany).

### 3.2. Plant Material and Extract Preparation

Ripe fruits of black chokeberry (*Aronia melanocarpa* (Michx.)) and bilberry (*Vaccinium myrtillus* L.) originating from Zagreb’s surrounding area were purchased at the local market. The plant samples were authenticated by the Department of Pharmacognosy, Faculty of Pharmacy and Biochemistry (University of Zagreb, Zagreb, Croatia). Fresh berries (10.00 g) were macerated with 50% ethanol (100 mL) for 24 h. After maceration, samples were extracted for an additional 15 min in an ultrasonic bath and filtered through Whatman paper No. 1, using a Büchner funnel. The ethanol was removed in a rotary evaporator. The residues were freeze-dried and used for further studies. The extraction yields for black chokeberry and bilberry were 29.56% and 26.32%, respectively.

### 3.3. TLC Analysis

Phenolic compounds were detected by thin-layer chromatography. Aliquots (10 μL) of the freeze-dried hydroethanolic extracts and standards were applied to a precoated silica gel 60 F254 TLC plate and developed in glass chambers previously saturated with the mobile phases: ethyl acetate—formic acid—acetic acid—water (100:11:11:27, *v*/*v*/*v*/*v*). The chromatograms obtained were dried in a stream of air for a few minutes. The flavonoids and phenolic acids were analyzed under UV light at 365 nm after spraying with a natural products-polyethylene glycol reagent (1% methanolic solution of 2-aminoethyl diphenylborinate and 5% ethanolic solution of PEG 4000 [81].

### 3.4. Total Polyphenols Determination

Total polyphenol content was determined according to the procedure described by Feng et al. [82], with slight modifications. Briefly, 0.5 mL of the freeze-dried hydroethanolic extracts or standard of the appropriate concentration was added to the volumetric flask and mixed with 1 mL of the Folin-Ciocalteau’s phenol reagent, 10 mL of distilled water, and made up to a volume of 25 mL with 10% sodium carbonate. Samples were shaken well and stored in the dark at room temperature for 30 min before absorbance was measured at 760 nm. Gallic acid was used to generate a calibration curve between 50 and 600 µg/mL at 8 concentration levels. Results were expressed as gallic acid equivalents (GAE) per 100 g of sample.

### 3.5. Determination of Hydroxycinnamic Acid Derivatives

Hydroxycinnamic acid derivatives were determined according to the European Pharmacopoeia [83] with slight modifications. Freeze-dried hydroethanolic extracts (0.20 g) were dissolved in 50% ethanol (50 mL). An aliquot of the extract (1.0 mL) was mixed with 0.5 M hydrochloric acid (2 mL), Arnow’s reagent (10% aqueous solution of sodium nitrite and sodium molybdate, 2 mL), and 8.5% sodium hydroxide (2 mL) and diluted with water to 10.0 mL. The absorbance was measured at 525 nm against the blank sample. The percentage of total hydroxycinnamic acid content was calculated and expressed as chlorogenic acid according to the equation (%) = A × 2.65/m. A represents the absorbance of the test solution while *m* represents the mass of the extract in grams. 

### 3.6. Total Anthocyanins Determination

The anthocyanin content was determined according to the European Pharmacopoeia [83] with slight modifications. Briefly, 0.05 g of the freeze-dried hydroethanolic extracts were dissolved in water (1 mL). The solution was then diluted 50-fold with 0.1% of hydrochloric acid. The absorbance of the solution was measured at 528 nm against 0.1% hydrochloric acid as a blank. The content of anthocyanins was expressed as cyanidin 3-*O*-glucoside chloride according to the equation (%) = A ×50/718 × m. A represents the absorbance of the test solution, m represents the mass of the extract in grams, and 718 represents the specific absorbance of cyanidin 3-*O*-glucoside chloride at 528 nm.

### 3.7. Total Flavonoids Determination

The flavonoid content was determined spectrophotometrically according to the method described in the European Pharmacopoeia [83]. Freeze-dried hydroethanolic extracts (0.600 g) were mixed with 1.0 mL of hexamethylenetetramine (5 g/L), 20 mL of acetone, and 2.0 mL of hydrochloric acid (250 g/L) and heated in a water bath under reflux for 30 min. After cooling, the extracts were filtered through cotton, and the residue was extracted twice with 20 mL of acetone for 10 min and diluted to 100 mL with acetone. The obtained extract was mixed with 20 mL of distilled water and extracted with ethyl acetate. The ethyl acetate fractions were washed with distilled water, filtered over anhydrous sodium sulphate, and diluted with ethyl acetate to 50.0 mL. An aliquot (10.0 mL) was mixed with 1.0 mL of an aluminum chloride solution and diluted to 25.0 mL with a 5% methanolic solution of acetic acid. The absorbance of the test solution was measured after 30 min at 425 nm. Total flavonoids were expressed as isoquercitrin and calculated according to the following equation: (%) = A × 1.25/m. A represents the absorbance of the test solution, while *m* represents the mass of the extract in grams.

### 3.8. Determination of Phenolic Acids and Flavonoids by High-Performance Liquid Chromatography

Identification and quantification of phenolic acids and flavonoids in freeze-dried ethanolic extracts were performed using an Agilent 1260 Infinity II liquid chromatograph equipped with an autosampler, quaternary pump, column oven, and DAD detector (Agilent Technologies, Santa Clara, CA, USA) according to the method described previously [84]. The analysis was performed using Zorbax Eclipse XDB-C18 column (4.6 × 250 mm, particle size 5 µm, Agilent Technologies). The mobile phase consisted of 2% (*v*/*v*, A) formic acid and acetonitrile (B). Elution was performed with the following gradient: 100–91% A from 0 to 12 min, 91–87% A from 12 to 20 min, 87–67% A from 20 to 30 min, 67% A from 30 to 32 min, 67–57% A from 32 to 42 min, 57% A from 42 to 60 min, 57–100% A from 60 to 60.50 min and 100% A from 60.50 to 73 min at a flow rate of 0.8 mL/min and a column temperature of 40 °C. Samples were prepared by dissolving in 50% EtOH to give a concentration of 30 mg/mL, while the concentration of standards was 2 mg/mL. Samples were filtered through a 0.45 µm syringe filter. Peaks were identified by comparing retention times and UV/VIS spectra with those of the standard solution. Chlorogenic and neochlorogenic acid were quantified at 330 nm using calibration curves obtained by linear regression analysis of six calibration points, y = 3120x + 376.9, R^2^ = 0.9992 for chlorogenic acid and y = 3433.3x + 326.85, R^2^ = 0.9977. Rutin, hyperoside and isoquercitrine were quantified at 240 and 360 nm using calibration curves obtained by linear regression analysis of six calibration points. The calibration curves for rutin, hyperoside and isoquercitrine were y = 947.11 − 28.683, R^2^ = 0.9949; y = 2115x − 4.5056, R^2^ = 0.99998; y = 1419.4x + 15.089, R^2^ = 1. Results were expressed as mg of phenolic acid or flavonoid per g of extract. 

### 3.9. Src Tyrosine Kinase Inhibition Assay 

To evaluate the inhibition of Src tyrosine kinase (Src), a time-resolved fluorescence resonance energy transfer (TR-FRET) tyrosine kinase assay was used according to the procedure described by Jelić et al. [85]. The assay was performed using a low-volume 384-well plate (Storplate-384-deep-well-V plate, Perkin Elmer, Waltham, MA, USA). The mother plate contained samples dissolved in DMSO, which were serially diluted 1:3 using an automated Janus pipetting workstation (Janus Integrator Platform, 8 tips, AJ8001, Perkin Elmer). Samples (100 nL) were then transferred to the test plate using a nanovolume dispenser (Mosquito 3019-002, TTP Labtech, Melbourn, UK). In the first step, the kinase reaction was started by mixing 0.5 nM Src (5 µL) with a combination of 50 nM peptide substrate (2.5 µL) and 200 µM ATP (2.5 µL). After three hours of incubation at 20 ± 2 °C, the mixture was diluted in kinase buffer containing 50 mM HEPES, 1 mM EGTA, 10 mM MgCl_2_, 2 mM DTT, and 0.01% Tween 20. Samples were analyzed at a concentration range of 0.02–1000 µg/mL. In the second step, 10 mM EDTA (5 µL) and 1 nM of Europium-labeled anti-phospho antibodies (5 µL) prepared in LANCE detection buffer were added and then incubated at 20 ± 2 °C for 3 h. TR-FRET signal was recorded at Ex340/Em615-665 using an EnVision plate reader (XciteMultilabel Reader, 2104-0020, Perkin Elmer). The final concentration of DMSO was 1%. Staurosporine was used as a reference control substance.

### 3.10. DPPH Radical-Scavenging Assay

The DPPH radical-scavenging assay was performed according to the method described by Harput et al. [86] with minor modifications. Appropriate serial dilutions of the freeze-dried hydroethanolic extracts and chlorogenic acid (800–6.25 µg/mL) in 50% EtOH were prepared in 96-well plates. Freshly dissolved 0.36 mM DPPH^•^ (70 µL) was added to the 130 µL of a sample, and the reaction mixture was shaken vigorously and incubated in the dark for 30 min. The absorbance was measured at 492 nm using a microplate reader (Chromate, Palm City, FL, USA). The radical-scavenging activity was determined by comparing the absorbance with that of the blank (100%) containing only DPPH^•^ and solvent. 

### 3.11. NO Radical-Scavenging Assay

The radical-scavenging activity of nitric oxide was investigated according to the method described by Jing et al. [87] with slight modifications. To determine the NO radical-scavenging activity of the freeze-dried hydroethanolic extracts and chlorogenic acid, 80 µL of the serially diluted samples (800–6.25 µg/mL) were added to the 96-well plate and 80 µL of 2 mM sodium nitroprusside, dissolved in PBS (0.01 mM, pH 7.4), was added to each well. The plate was incubated under light at room temperature for 120 min. After incubation, 40 µL of 1% sulphanilamide in phosphoric acid was added and held for 5 min. Finally, 40 µL of 0.1% NED was added and the absorbance was immediately measured at 545 nm on the microplate reader (Chromate, Palm City, FL, USA). The NO radical-scavenging activity was calculated by comparing the absorbance with that of the blank (100%) containing all reagents except the tested sample.

### 3.12. Statistical Analysis

All experiments were performed in triplicate and results are expressed as mean ± standard deviation. The SC_50_ and IC_50_ values were calculated by linear regression extrapolation. Differences between the obtained results were determined using the ordinary one-way ANOVA and post hoc Tukey’s multiple comparisons tests. Pearson’s correlation coefficient was calculated to determine the degree of association between the two variables. All statistical analyses were performed using GraphPad Prism software (version 8.4.3) and Microsoft Excel (Microsoft office 365). All values with *p* < 0.05 were considered statistically significant.

## 4. Conclusions

As far as we know, this study is the first to demonstrate the inhibitory properties of black chokeberry and bilberry fruits on Src tyrosine kinase activity. The hydroethanolic fruit extracts studied significantly suppressed Src activity. The inhibitory effect was comparable in both and was mainly due to the presence of chlorogenic acid. These extracts also showed a strong ability to scavenge NO radicals, with black chokeberry being a more potent antioxidant. Our results contribute to the clarification of the complex mechanisms of the anticancer and anti-inflammatory effects of black chokeberry and bilberry, in which phenolic components play an important role. In particular, the current study highlights the importance of hydroxycinnamic acids, especially chlorogenic acid, which has great potential to become a leading component in the discovery of new anticancer and anti-inflammatory drugs. In addition, further research is needed to adequately support the use of black chokeberry and blueberry fruits in the prevention and treatment of diseases associated with oxidative stress and chronic inflammation. 

## Figures and Tables

**Table 1:** Content of total polyphenols, hydroxycinnamic acids, anthocyanins, and flavonoids (%, g/100 g of extract) in black chokeberry and bilberry fruit extracts.

Sample	Polyphenols	Hydroxycinnamic Acids	Anthocyanins	Flavonoids
Black chokeberry	5.90 ± 0.18 ^a^	4.09 ± 0.13 ^a^	0.41 ± 0.02 ^b^	0.17 ± 0.003 ^a^
Bilberry	4.96 ± 0.09 ^b^	1.51 ± 0.22 ^b^	1.06 ± 0.01 ^a^	0.12 ± 0.002 ^b^

The data are expressed as mean values of three independent experiments ± standard deviation. Mean values displaying different letters (a, b) within each column are significantly different according to Tukey’s multiple comparisons test at confidence level *p* < 0.05.

**Table 2:** Content of phenolic acids and flavonoids (mg/g of extract) of black chokeberry and bilberry extracts determined by the HPLC-DAD method.

Compound	Rt (min)	Black Chokeberry	Bilberry
Neochlorogenic acid	14.12	2.07 ± 0.13	n.d.
Chlorogenic acid	18.45	2.05 ± 0.00 ^a^	2.54 ± 0.02 ^b^
Rutin	27.58	0.34 ± 0.01	n.d.
Hyperoside	28.42	0.50 ± 0.03 ^a^	0.26 ± 0.01 ^b^
Isoquercitrin	28.71	0.67 ± 0.01 ^a^	1.36 ± 0.01 ^b^

The data are expressed as mean values of three independent experiments ± standard deviation. Mean values displaying different letters (a, b) within each row are significantly different according to Tukey’s multiple comparisons test at confidence level *p* < 0.05. n.d.—not detected.

**Table 3:** DPPH radical-scavenging activity (%) of black chokeberry and bilberry fruit extracts in comparison with chlorogenic acid.

Sample	6.25 µg/mL	12.5 µg/mL	25 µg/mL	50 µg/mL	100 µg/mL	200 µg/mL	400 µg/mL	800 µg/mL
Black chokeberry	8.43 ± 1.93 ^b^	15.56 ± 1.44 ^b^	19.44 ± 8.08 ^b^	32.98 ± 2.78 ^b^	47.73 ± 2.13 ^b^	55.12 ± 4.40 ^b^	75.72 ± 3.51 ^b^	91.85 ± 3.11 ^a^
Bilberry	6.76 ± 1.14 ^b^	9.09 ± 1.82 ^c^	13.80 ± 1.70 ^b^	20.76 ± 2.90 ^c^	35.38 ± 2.07 ^c^	43.11 ± 0.02 ^c^	57.24 ± 1.53 ^c^	84.84 ± 0.71 ^b^
Chlorogenic acid	20.87 ± 2.18 ^a^	36.65 ± 1.36 ^a^	65.52 ± 1.37 ^a^	77.66 ± 3.97 ^a^	91.01 ± 3.98 ^a^	95.03 ± 0.93 ^a^	95.24 ± 1.18 ^a^	95.62 ± 1.53 ^a^

The data are expressed as mean values of three independent experiments ± standard deviation. Mean values displaying different letters (a–c) within each column are significantly different according to Tukey’s multiple comparisons test at confidence level *p* < 0.05.

**Table 4:** NO radical-scavenging activity (%) of black chokeberry and bilberry fruit extracts in comparison with chlorogenic acid.

Sample	6.25 µg/mL	12.5 µg/mL	25 µg/mL	50 µg/mL	100 µg/mL	200 µg/mL	400 µg/mL	800 µg/mL
Black chokeberry	15.64 ± 0.23 ^b^	22.95 ± 5.52 ^b^	25.36 ± 2.90 ^b^	32.53 ± 1.45 ^b^	39.12 ± 0.60 ^b^	56.82 ± 2.73 ^b^	80.98 ± 2.60 ^a^	94.28 ± 1.38 ^a^
Bilberry	2.12 ± 0.26 ^c^	4.44 ± 3.02 ^c^	7.39 ± 5.08 ^c^	10.37 ± 4.03 ^c^	20.58 ± 1.29 ^c^	34.14 ± 3.31 ^c^	55.02 ± 2.83 ^b^	62.78 ± 0.48 ^c^
Chlorogenic acid	24.94 ± 1.61 ^a^	37.78 ± 2.99 ^a^	57.39 ± 0.50 ^a^	64.88 ± 1.42 ^a^	68.65 ± 1.01 ^a^	73.57 ± 1.90 ^a^	77.44 ± 0.61 ^a^	78.16 ± 1.72 ^b^

The data are expressed as mean values of three independent experiments ± standard deviation. Mean values displaying different letters (a–c) within each column are significantly different according to Tukey’s multiple comparisons test at confidence level *p* < 0.05.

**Table 5:** Comparative SC_50_ values (µg/mL) of black chokeberry and bilberry fruit extracts and chlorogenic acids obtained for DPPH and NO radical-scavenging activity.

Sample	DPPH^•^ Scavenging Activity	NO^•^ Scavenging Activity
Black chokeberry	153.3 ± 13.2 ^b^	161.1 ± 11.4 ^b^
Bilberry	298.8 ± 16.2 ^a^	352.2 ± 26.1 ^a^
Chlorogenic acid	18.4 ± 0.4 ^c^	20.4 ± 0.3 ^c^

The data are expressed as mean values of three independent experiments + standard deviation. Mean values displaying different letters (a–c) within each column are significantly different according to Tukey’s multiple comparisons test at confidence level *p* < 0.05.

## Data Availability

Not applicable.
